# Forecasting ESKAPE infections through a time-varying auto-adaptive algorithm using laboratory-based surveillance data

**DOI:** 10.1186/s12879-014-0634-9

**Published:** 2014-12-06

**Authors:** Antonio Ballarin, Brunella Posteraro, Giuseppe Demartis, Simona Gervasi, Fabrizio Panzarella, Riccardo Torelli, Francesco Paroni Sterbini, Grazia Morandotti, Patrizia Posteraro, Walter Ricciardi, Kristian A Gervasi Vidal, Maurizio Sanguinetti

**Affiliations:** Arkegos International Study Centre, Rome, Italy; Institute of Public Health (Section of Hygiene), Università Cattolica del Sacro Cuore, Rome, Italy; Advanced Research Centre for Applied Science, Rome, Italy; Institute of Microbiology, Università Cattolica del Sacro Cuore, 00168 Largo F. Vito 1, Rome, Italy; Clinical Laboratory, Ospedale San Carlo, Rome, Italy; IMT Institute for Advanced Studies, Lucca, Italy

**Keywords:** ESKAPE infections, Clinical microbiology laboratory data, Time series analysis, TVA algorithm, Forecasting

## Abstract

**Background:**

Mathematical or statistical tools are capable to provide a valid help to improve surveillance systems for healthcare and non-healthcare-associated bacterial infections. The aim of this work is to evaluate the **t**ime-**v**arying **a**uto-adaptive (TVA) algorithm-based use of clinical microbiology laboratory database to forecast medically important drug-resistant bacterial infections.

**Methods:**

Using TVA algorithm, six distinct time series were modelled, each one representing the number of episodes per single ‘ESKAPE’ (***E****nterococcus faecium*, ***S****taphylococcus aureus*, ***K****lebsiella pneumoniae*, ***A****cinetobacter baumannii*, ***P****seudomonas aeruginosa* and ***E****nterobacter* species) infecting pathogen, that had occurred monthly between 2002 and 2011 calendar years at the Università Cattolica del Sacro Cuore general hospital.

**Results:**

Monthly moving averaged numbers of observed and forecasted ESKAPE infectious episodes were found to show a complete overlapping of their respective smoothed time series curves. Overall good forecast accuracy was observed, with percentages ranging from 82.14% for *E. faecium* infections to 90.36% for *S. aureus* infections.

**Conclusions:**

Our approach may regularly provide physicians with forecasted bacterial infection rates to alert them about the spread of antibiotic-resistant bacterial species, especially when clinical microbiological results of patients’ specimens are delayed.

**Electronic supplementary material:**

The online version of this article (doi:10.1186/s12879-014-0634-9) contains supplementary material, which is available to authorized users.

## Background

Despite advances in diagnostic, therapeutic and vaccination countermeasures, infectious diseases still are one of major challenges worldwide [1], which engage a lot of biomedical research and public health efforts to understand, treat, control and prevent them [2]. In particular, infections caused by antibiotic-resistant bacteria such as the ‘ESKAPE’ pathogens (***E****nterococcus faecium*, ***S****taphylococcus aureus*, ***K****lebsiella pneumoniae*, ***A****cinetobacter baumannii*, ***P****seudomonas aeruginosa* and ***E****nterobacter* species), which are effectively capable of ‘escaping’ the biocidal action of antimicrobials, continue to rise and cause significant morbidity and mortality [3].

This makes the management of bacterial infections very difficult not only for hospitalized patients but also otherwise healthy non-hospitalized patients [4],[5] and, in the same time, poses the need for continuously re-evaluating global infectious disease surveillance systems [6]. To support infectious disease and/or infection control specialists locally, computerized data on the isolation of clinically relevant microbial species and their drug-resistance profiles are usually available from microbiology laboratory information systems. Thus, the patients’ outcomes may be optimized by adequate initial antibiotic therapy that would be selected on the basis of local resistance patterns [2], although it should also be important to improve the ability to predict likely infecting pathogens when empirical therapies need to be prescribed [4].

As surveillance data are often measured repeatedly at equal intervals of time, autoregressive integrated moving average (ARIMA) models, also termed Box-Jenkins models [7], which use time series analyses [8], appear to be powerful tools to monitor and predict the incidence of several infectious diseases, including dengue [9], malaria [10], haemorrhagic fever with renal syndrome [11] and hepatitis E [12], as well as the influenza-related mortality [13]. Furthermore, by using an extension of the ARIMA method called transfer function [8], a temporal relationship between antimicrobial use and resistance was demonstrated for the ceftazidime and Gram-negative bacilli and the imipenem and *P. aeruginosa* combinations [14]. Employing ARIMA or autoregressive moving average (ARMA) models for time series forecasts has become increasingly popular, but the major limitation with their use is the pre-assumed linearity of the models [15], that often leads to combining them with other statistical techniques [12].

The objective of this study was to evaluate a time series method using a **t**ime-**v**arying **a**uto-adaptive (TVA) algorithm for forecasting drug-resistant bacterial infections, through use of the data collected in a large Italian hospital microbiology laboratory. By this approach, we were able to predict the frequency on a monthly basis of single ESKAPE infectious episodes in inpatient or outpatient healthcare settings.

## Methods

### Study setting

General Hospital ‘Agostino Gemelli’ from the Università Cattolica del Sacro Cuore (Rome, Italy) is a 1500-bed tertiary care facility, which comprises a full range of medical and surgical specialties, a paediatric unit, a maternity unit, a cardiovascular surgery unit and four (general, neonatal, paediatric and post-surgical) intensive care units. It was opened in 1964 as the referral hospital for a healthcare area of approximately 200 000 inhabitants, and in 2004 it was enlarged to enclose a multifunctional platform, in which are housed centralized diagnostic laboratories, operating rooms and the Emergency Department. The entire structure admits ~50 000 patients per year.

In 1998, a multidisciplinary team, composed of a small, technically focused, clinical group of microbiologists, hygienists, epidemiologists and physicians, was formed with tasks of the infection prevention and control and, recently, the antimicrobial stewardship. Members of this group share the reports on local microbial ecology data, which are collected at the hospital level (see below) in order to adapt patient care to the infection risk.

### Microbiological data set

This study was conducted using local data that were exported daily from the clinical microbiology laboratory information system, by means of VIGIguard™ Active Surveillance Epidemiology software (bioMérieux Diagnostics Search, Marcy l’Étoile, France), and were reported into a customized database. Data included the patient identifiers, hospital wards or outpatient services, types of specimen, species of isolates and antimicrobial susceptibility patterns of the isolates. With respect to the last-named data, minimum inhibitory concentrations were determined and interpreted according to the Clinical and Laboratory Standards Institute breakpoints [16]. Duplicate or multiple isolates were disregarded, and only the first one of each species per patient was maintained into the database and used for our analysis. With regards to the specimen sources, bacterial isolates were recovered from non-invasive (lower respiratory tract or urine) and invasive (blood or cerebrospinal fluid) specimens.

### Study design, time series and forecasting algorithm

This study was ecologically designed, and utilized no patients’ identities (names and hospital codes) or personal information. Overall microbiology laboratory data were retrieved from January 2002 to July 2011, and those with respect to the six ESKAPE microorganisms were extracted and their daily numbers, summed per month, were used for one-step-ahead forecasting purposes. Thus, data utilized for the analysis included monthly time series of isolates of *E. faecium*, *S. aureus*, *K. pneumoniae*, *A. baumannii*, *P. aeruginosa* and *Enterobacter* species, all of which representing single drug-resistant infectious episodes. The first 36 months of data (January 2002 to December 2004) were used as the training set, whereas all remaining data were used to evaluate the forecasting method. We computed optimal single-series forecasts for the ESKAPE infection occurrence, using a univariate method where forecasts are dependent only on present and past values of the single series being forecasted, possibly augmented by a function of time such as a linear trend [17]. For each time series, forecast accuracy was assessed at the horizon of 30 days in advance, which, to our view, would reflect the ongoing nature of healthcare surveillance policies. Data from each time series were treated as individual time series and analysed and evaluated separately. No present and past values of other (predictor or explanatory) variables were assessed.

Before our attempt to forecast each time series, we conducted preliminary descriptive analyses of the data to identify relevant features, such as autocorrelation, seasonal patterns, trend, outliers and any other notable fluctuations, in the series. Also, we evaluated whether or not each time series was stationary (i.e., whether or not basic statistical properties such as the mean and variance of the series remained constant through time). Initial data analysis was conducted via the visualization of time plots and correlograms and the computation of basic descriptive statistics.

As it is known, a forecasting method may arise from identifying a particular model for the given data and finding optimal forecasts conditional on that model, or it may simply be an algorithmic rule and need not depend on an underlying probability model [17]. A detailed description of our forecasting method that uses a TVA algorithm was reported previously, although it has been otherwise applied [18]. Here, we recall the essence of this algorithm, which is derived from an original physics-based theoretical approach, i.e., every physical phenomenon is described by a characteristic time parameter that is valid for the temporal interval during which it is observed [18]. Consequently, if *f*(*x*, *t*) represents a certain time-dependent observation, its average value must be transformed as follows:

$$\lim_{T\to \infty }\frac{1}{T}\int_{0}^{T}f\left(x,t\right)dt\to \int_{t-T\left(t\right)}^{t}f\left(x,t\right)dx$$

where *T*(*t*) represents the characteristic time parameter that, in turn, depends on the phenomenon *f*(*x*, *t*) observed in a defined time window. As *T*(*t*) can be applied to time series analysis, TVA algorithm allows to determine, for each point of the time series, a *T*(*t*) value that is able to forecast the next value [18].

For each time series, an autocorrelation function graph was obtained to assess whether the observations showed a short- or long-time dependence, or whether they showed a seasonal pattern, in accordance with the formula:

$$r_{k}=\frac{\sum_{t=1}^{N-k}\left(x_{t}-\bar{x}\right)\left(x_{t+k}-\bar{x}\right)}{\sum_{t=1}^{N}{\left(x_{t}-\bar{x}\right)}^{2}}$$

where *x*_*t*_ indicates the point of the time series considered at time *t*; *r*_*k*_ expresses the degree of correlation between the value detected at time *t* and the value detected at *t* + *k*, that is, *x*_*t*+*k*_; *N* indicates the total number of infections in the series analysed; and $\bar{x}$ represents the average value of the series calculated on the *N*. As each point *x*_*t*_ of the series is typically composed of three components, that is, seasonal (*S*_*t*_), trend (*T*_*t*_), and casual (*U*_*t*_), according to *x*_*t*_ = *S*_*t*_ + *T*_*t*_ + *U*_*t*_, the time moving average (MA) was chosen as a filter to remove the component *U*_*t*_, thus maintaining unchanged the other components, in a sub-interval *Q* of *N* infections according to the equation ${\bar{x}}_{k}=\frac{1}{Q}\sum_{t=k-Q}^{k}x_{t}$ where *Q* is a function of the single series analysed.

The forecast of monthly infections was carried out on both the original, not filtered series {*x*_*k*_} and the series after application of the time filters $\left\{{\bar{x}}_{k}\right\}$. In the former case, the value’s tendency was derived by the inverse of above equation, that is, ${\hat{x}}_{k}=Q{\bar{x}}_{k}^{*}-\sum_{t=k-Q}^{k-1}x_{t}$ where ${\bar{x}}_{k}^{*}$ is the forecast produced at step *k* for the average time series $\left\{{\bar{x}}_{k}\right\}$. To assess forecast accuracy, we also computed the mean absolute error (MAE) and the mean absolute percentage error (MAPE) parameters, according to the following equations:

$MAE=\frac{1}{N}\sum_{k=0}^{N-1}\left|V_{k+1}-F_{k}\right|\;\mathrm{MAPE}=\frac{1}{N}\sum_{k=0}^{N-1}100\left|\frac{V_{k+1}-F_{k}}{V_{k+1}}\right|$ where *F*_*k*_ represents the forecasting value, calculated at step *k*, of the series $\left\{{\bar{x}}_{k}\right\}$ for the value *V* recorded at step *k* +1, that is, *V*_*k* + 1_. A lower MAPE value indicates a better fit of the time series data.

### Ethical review

The present study was reviewed by the institutional review committee of the Università Cattolica del Sacro Cuore, and it was found that utilization of clinical laboratory surveillance data did not require oversight by an ethics committee.

## Results

### Data characteristics

A total of 33 185 non-duplicate bacterial isolates, that were found to be *in vitro* resistant to one or more antimicrobials, were obtained from single infectious episodes of inpatients and outpatients between January 2002 and July 2011, as reported into the clinical microbiology laboratory database. As accounting for 92.6% of above episodes, *Escherichia coli* (29.6%), *P. aeruginosa* (15.6%), *S. aureus* (13.1%), *A. baumannii* (12.4%), *K. pneumoniae* (6.9%), *Proteus mirabilis* (4.1%), *Enterobacter* species (3.9%), *Stenotrophomonas maltophilia* (3.6%) and *E. faecium* (3.4%) were the most frequently isolated species during the study time period. Among drug-resistant ESKAPE isolates, 75.9% of *E. faecium* isolates were resistant to vancomycin, 91.7% of *S. aureus* isolates to methicillin, 95.0% of *K. pneumoniae* isolates to extended-spectrum cephalosporins, 80.8% of *A. baumannii* isolates to carbapenems, 47.3% of *P. aeruginosa* isolates to carbapenems and extended-spectrum cephalosporins, and 85.6% of *Enterobacter* species (*E. cloacae*, *E. aerogenes*, *E. agglomerans* and *E. sakazaki*) isolates to extended-spectrum cephalosporins. Therefore, starting from the original database, we chose to obtain 6 distinct ESKAPE time series, each one corresponding to the number of episodes per single infecting species that had occurred monthly during the 9-year time period.

### TVA algorithm for ESKAPE infection forecasting

**Figure 1:**
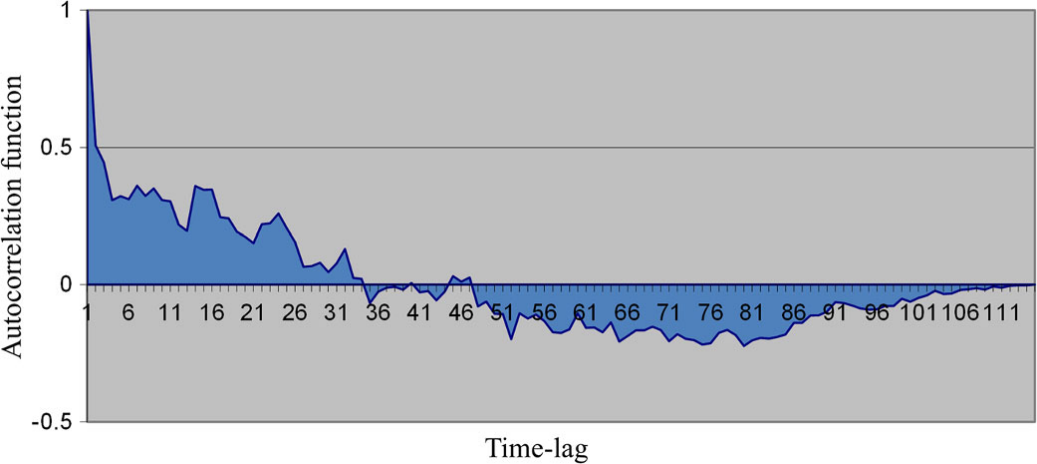
**Correlogram of the time series of drug**-**resistant** ***E. faecium*** **infections observed between January 2002 and July 2011.** Autocorrelations were computed for data values at varying time lags.

**Figure 2:**
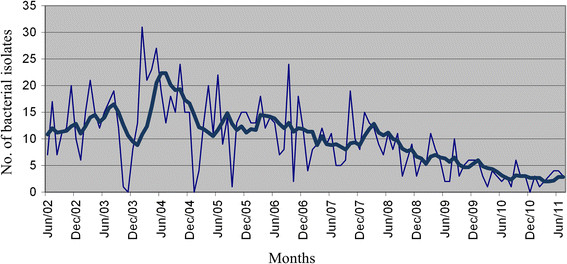
**Observed and moving averaged numbers of drug**-**resistant** ***E. faecium*** **infectious episodes during the 9**-**year study period.** The smoothed time series, represented in bold, was obtained using a 3-month moving average transformation.

Autocorrelation analysis of these monthly aggregated data revealed that each of individual ESKAPE infection series exhibited randomness or, at least, a behaviour consistent with the presence of short-term correlation between an infectious episode and the next ones. A representative correlogram is depicted in Figure 1, showing that one or more of autocorrelations were significantly non-zero. To reduce autocorrelation to white noise, time MAs were used for all of ESKAPE infections. Thus, we plotted the smoothed frequencies of bacterial isolates that were derived by a time MA transformation, i.e., the value plotted for a specific month was the average of the value observed that month, the previous month(s) and the next month(s). Figure 2 shows an example of the smoothed series plots obtained for *E. faecium*, *S. aureus*, *K. pneumoniae*, *A. baumannii*, *P. aeruginosa* and *Enterobacter* species infections, by using 3-, 4-, 6- or 12-month MAs as appropriate.

Therefore, a forecasting TVA algorithm was built starting from the hypothesis that each value within a generic time series is influenced by the values occurred previously and that it, in turn, will influence future values. After a random-span time window was defined *a priori*, the time series values were clustered, and to each cluster was associated a value derived from computing the probability function. The resulting cluster values were ordered and the first ranking number was considered as the most likely value for the next trial.

**Figure 3:**
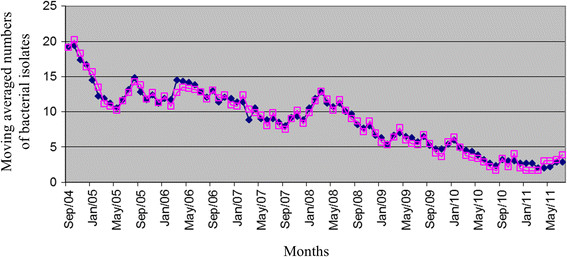
**Monthly moving averaged numbers of observed** **(black symbols)** **and forecasted** **(purple symbols)** **drug**-**resistant** ***E. faecium*** **infectious episodes.** A complete overlapping between the smoothed series curves was observed for the entire study period (years 2002–2011). Shown is, for convenience, the interval time between September 2004 and May 2011.

Using the TVA algorithm, we predicted the monthly numbers of drug-resistant infections caused by each of ESKAPE bacteria during the study period. Graphical representations of the results, of which an example is given in Figure 3, show plots of observed frequencies that overlapped those of forecasted frequencies. Table 1 summarizes the TVA algorithm forecasting performances obtained with all the ESKAPE time series analysed. As it can see, an overall good forecast accuracy was achieved, with percentages ranging from a value of 82.14% for *E. faecium* infections to a value of 90.36% for *S. aureus* infections.

**Table 1:** **Monthly forecasting of** “**ESKAPE**” **infections**: **assessment parameters and performance of the time**-**varying auto**-**adaptive algorithm**

Bacterial species	No. of recorded infections (years 2002– 2011)	Time MA (months) ^a^	MAPE ^b^	Accuracy rate (%)
*E. faecium*	1,142	3	9.22	82.14
*S. aureus*	4,332	4	5.15	90.36
*K. pneumoniae*	2,284	12	3.09	89.61
*A. baumannii*	4,106	12	3.18	84.93
*P. aeruginosa*	5,165	3	4.94	87.95
*Enterobacter* species	1,293	6	7.23	84.34

Abbreviations: MA, moving average; MAPE, mean absolute percentage error.

^a^The time MA was identified using the autocorrelation function graph, as detailed in Methods.

^b^MAPE value was calculated based on observed values and fitted values from 2002 to 2011.

## Discussion

As a widely applicable, multidisciplinary science, forecasting is an important activity for statisticians, economists, operational researchers, management scientists and decision scientists, as well as it has become an essential that drives decision-making in many fields of economic, industrial and scientific planning [17]. In the healthcare setting, forecasting has been explored as a method to improve emergency department services, where accurate forecasts of demand can guide the allocation of human and physical resources to allow an efficient patient flow [19],[20]. This, in turn, may minimize patient care delays and improve the overall quality of care. Likewise, emergence and re-emergence of infectious diseases with pandemic potential has led to growing interest in their analysis [21], so now a large amount of infectious disease data is routinely collected by laboratories, healthcare providers and government agencies in an effort to prevent, detect and manage infectious diseases outbreaks. In this context, one-step-ahead forecasts, especially when syndromic information is incorporated into the forecasting model, can be used to detect high-risk areas for outbreaks and, consequently, to develop efficient targeted surveillance [22].

While the time series analysis is used to extract meaningful statistics and other characteristics of data, time series forecasting is able to predict future values of the series based on its historical values. However, with time-series data, the modelling process is complicated by the need to model not only the interdependence between the series, but also the serial dependence within the component series [17]. Several technical resources are available to guide analysts in building and interpreting correlation models [23],[24], as well as review articles [8],[25],[26] and biomedical examples are also available [27],[28]. Thus, good forecasting depends on finding a suitable model for a given time series but, despite a plenty of software available to make it easy to fit the class of linear stochastic processes, namely ARIMA models, it is still difficult to know when to use an ARIMA model and how to choose which ARIMA model to use. ARIMA models have long been applied in various medical specialties [8],[29], until to recently predict the infectious disease incidence due to its structured modelling basis and acceptable forecasting performance [9]-[12]. However, obtaining an ARIMA model that closely fit a type of time series data requires that different ARIMA models are simultaneously constructed and checked for their goodness-of-fit prior to reach the satisfactory final model [11],[12],[14]. To this regard, it is noteworthy that an artificial neural network [17] was used in combination with an ARIMA model to take into account the linear and nonlinear behaviours of time series data, in order to forecast hepatitis E infections in Shanghai [12]. Accordingly, in a recent comparison of the models’ forecasting accuracy, the multivariate seasonal ARIMA model (SARIMA), an expanded form of ARIMA, was shown to be the most appropriate for forecasting the number of patients admitted to the emergency department per day, as it was built to incorporate explanatory variables affecting that number [20].

The present study describes the development of TVA algorithm as a simple and reliable tool to predict future trends of drug-resistant ESKAPE infections. We noticed that the noise of each time series analysed (e.g., absence of periodicity, presence of instrumental errors and non-uniformity of measures) did not influence the TVA algorithm’s forecasting capability. Also, the TVA-algorithm forecast performance on filtered time series (i.e., purged of their casual components) was higher than 80%, as documented by MAPE measurements that gave good estimates of the actual time series (Table 1). Consistent with other studies [11],[14], MAs were here used as an easy and intuitive means, even though more sophisticated techniques, such as exponential and/or adaptive MAs, Kalman filters, Holt-Winter filters [17], would have to be employed to refine the forecast results. Thus, it is surprising that such a simple algorithm is capable of producing such good predictions, but this is possible because infectious episodes are outbreaks and, therefore, are self-exciting processes which would be expected to cluster at high values. How TVA algorithm performs as we go into the future it needs to be explored. To strengthen our findings, ARMA models of order (2,3), (1,3), (1,1), (2,1), (1,2) and (1,1) (the figures indicate autoregressive and moving average terms) were constructed using the training set 36-month data to provide adequate model fit for monthly ESKAPE infections due to *E. faecium*, *S. aureus*, *K. pneumoniae*, *A. baumannii*, *P. aeruginosa* and *Enterobacter* species, respectively. However, these models allowed forecast accuracies of 61.11% (*E. faecium*), 48.65% (*S. aureus*), 67.17% (*K. pneumoniae*), 73.02% (*A. baumannii*), 63.01% (*P. aeruginosa*) and 53.42% (*Enterobacter* species) (data not shown), that were much lower than those obtained using TVA algorithm (Table 1).

As ideally forecasts are an integral part of the planning system, and not a separate exercise [17], it is desirable that a relatively simple forecasting method, which is widely understood, can allow people who will actually use the forecasts (i.e., hospital epidemiologists) to suggest control action. Thus, while a forecast of an increasing death rate for a particular disease may lead to preventive action to try to reduce the spread of the disease [30], an abnormally high methicillin-resistant *S. aureus* (MRSA) infection rate at the hospital or unit level (i.e., medical intensive care unit) may lead to an education-based intervention to increase compliance with hand-disinfection procedures [31], or to abolish individual-level MRSA decolonization programs [32]. Alternatively, the forecast can be used as a target value [17]. In this sense, it may permit to continuously monitor, and eventually correct, hospital antimicrobial stewardship programs, that have proven highly successful in improving patient outcomes, reducing adverse events (including *Clostridium difficile*), reducing re-admission rates and even reducing antibiotic resistance [33].

Therefore, our method could be practically implemented in a clinical setting to provide attending physicians with forecasted rates of drug-resistant bacterial infections on a regular basis. This in order to alert them about the spread of bacterial species displaying resistance to one or more antimicrobials, and, in the meantime, to help them in the empirical prescription of antimicrobials when the microbiology (culture and/or susceptibility testing) results of clinical specimens are not yet available.

Our findings may have important clinical repercussions. The challenge of antimicrobial resistance continue to grow locally and globally, and this necessitates a significant shift in mind-set about the infection control, which is now considered to be vital to aid prevent the spread of resistant microorganisms [34]. To this regard, surveillance and feedback of results to clinicians is crucial to performance improvement in managing both healthcare- and non-healthcare-associated drug-resistant infections [35]. Therefore, surveillance data should possibly be accurate and consistent to effectively monitor trends and outbreaks, particularly for infections caused by MRSA, vancomycin-resistant enterococci and multidrug-resistant Gram-negative bacteria, including *Acinetobacter* and *Pseudomonas* species [36].

## Conclusion

Surveillance systems must include microbiology laboratory reporting of the isolation of clinically significant pathogens with relevant drug susceptibilities included [35], but it would also be advisable that the use of computer-based searches of laboratory records is supported from mathematical modelling and prediction, such as the TVA algorithm described here. While the success of such a method, when implemented, will greatly depend on accessible and regularly updated surveillance reports, further studies are yet needed to provide a large-scale evaluation of this potentially useful epidemiological tool.

## Authors’ original submitted files for images

Below are the links to the authors’ original submitted files for images. Authors’ original file for figure 1 Authors’ original file for figure 2 Authors’ original file for figure 3
