# Supplementary figures and images for: Forecasting ESKAPE infections through a time-varying auto-adaptive algorithm using laboratory-based surveillance data
Source: BMC Infect Dis. 2014 Dec 6;14:634. doi: 10.1186/s12879-014-0634-9 (PMC4266976; doi:10.1186/s12879-014-0634-9)

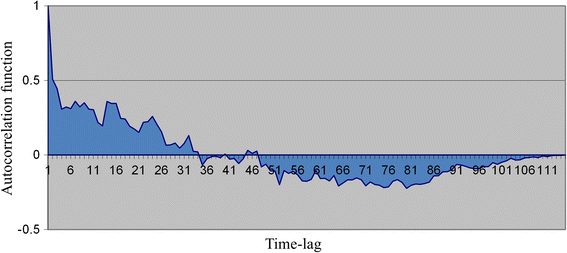

Supplement: Supplementary file 1 — Authors’ original file for figure 1 [file 12879_2014_634_MOESM1_ESM.gif]

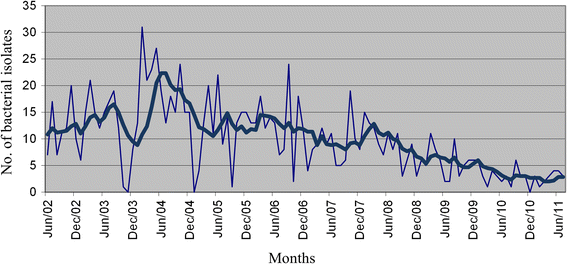

Supplement: Supplementary file 2 — Authors’ original file for figure 2 [file 12879_2014_634_MOESM2_ESM.gif]

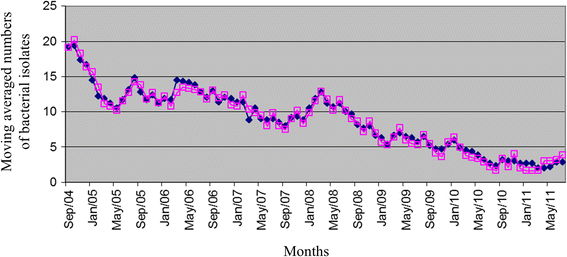

Supplement: Supplementary file 3 — Authors’ original file for figure 3 [file 12879_2014_634_MOESM3_ESM.gif]
